# Comprehensive view on genetic features, therapeutic modalities and prognostic models in adult T-cell lymphoblastic lymphoma

**DOI:** 10.1097/BS9.0000000000000114

**Published:** 2022-07-06

**Authors:** Qihua Zou, Shuyun Ma, Xiaopeng Tian, Qingqing Cai

**Affiliations:** a Department of Medical Oncology, Sun Yat-sen University Cancer Center, Guangzhou 510060, P. R. China; b State Key Laboratory of Oncology in South China, Collaborative Innovation Center for Cancer Medicine, Sun Yat-sen University Cancer Center, Guangzhou 510060, P. R. China

**Keywords:** Genetic features, Prognostic models, T-cell lymphoblastic lymphoma, Therapeutic modalities

## Abstract

Adult T-cell lymphoblastic lymphoma (T-LBL) is a rare and aggressive subtype of non-Hodgkin’s lymphoma that differs from pediatric T-LBL and has a worse prognosis. Due to its rarity, little is known about the genetic and molecular characteristics, optimal treatment modalities, and prognostic factors of adult T-LBL. Therefore, we summarized the existing studies to comprehensively discuss the above issues in this review. Genetic mutations of *NOTCH1/FBXW7*, *PTEN*, *RAS*, and *KMT2D*, together with abnormal activation of signaling pathways, such as the JAK-STAT signaling pathway were described. We also discussed the therapeutic modalities. Once diagnosed, adult T-LBL patients should receive intensive or pediatric acute lymphoblastic leukemia regimen and central nervous system prophylaxis as soon as possible, and cranial radiation-free protocols are appropriate. Mediastinal radiotherapy improves clinical outcomes, but adverse events are of concern. Hematopoietic stem cell transplantation may be considered for adult T-LBL patients with high-risk factors or those with relapsed/refractory disease. Besides, several novel prognostic models have been constructed, such as the 5-miRNAs-based classifier, 11-gene-based classifier, and 4-CpG-based classifier, which have presented significant prognostic value in adult T-LBL.

## 1. INTRODUCTION

T-cell lymphoblastic lymphoma (T-LBL) is an aggressive and rare subtype of non-Hodgkin lymphoma (NHL), which is a malignant tumor of thymic origin, occurs mainly in the mediastinum and other lymphoid organs with or without infiltration in bone marrow (BM), peripheral blood and cerebrospinal fluid.^1,2^

T-LBL is highly similar in morphology and immunophenotype to acute T-cell lymphoblastic leukemia (T-ALL) and was once considered to be the same disease.^2^ It was not until 1979 that T-LBL was first recognized as a distinct clinical type, and the main distinction from T-ALL is that T-LBL with BM infiltration <25%.^3^ And in recent years, molecular biology research has found that there is an essential difference between the 2 diseases.^4^

Until now, the standard therapeutic modalities for adult T-LBL have not been established, and the optimal modality of induction chemotherapy combined with consolidation treatment for adult T-LBL is controversial. Though event-free survival (EFS) and complete response (CR) rate have improved in T-LBL received intensive lymphoblastic leukemia (ALL)-like protocols,^5^ recurrence after CR remains high.^6,7^ The 5-year overall survival (OS) of relapsed T-LBL is merely 14%.^8^

Therefore, an in-depth understanding of the biological characteristics of T-LBL, elucidating the tumor heterogeneity and essential causes of the differential response to treatment could be significant for the accurate prognostic prediction and clinical decision-making for adult T -LBL.

## 2. MOLECULAR GENETIC FEATURES

### 2.1. Inherited susceptibility

Genetic predisposition of specific malignancies including T-LBL has been reported.^9^ A biallelic germline mutation in the *NBN* gene results in Nijmegen breakage syndrome (NBS).^10^ The gene encodes the nibrin protein, which is associated with repairment of DNA double-strand breaks. Patients with NBS have a propensity to develop T-LBL.^11^ Constitutional mismatch repair deficiency (CMMRD), resulting from a biallelic mutation of mismatch repair genes such as *MSH2*, *MSH6*, *MLH1*, or *PMS2*, is associated with the development of T-LBL.^11,12^

### 2.2. Genetic and epigenetic alterations

#### 2.2.1. *NOTCH1*/*FBXW7* mutations (N/Fmut).

NOTCH1 induces expression of the target gene by binding the active intracellular NOTCH1 (ICN1) to target gene transcriptional activators, which has a close association with the regulation of development, proliferation, and apoptosis in T cells.^13^ Additionally, FBXW7 is an E3 ubiquitin ligase complex, which binds to ICN1 to induce ubiquitination and degradation of proteasome. *FBXW7* mutations inhibit degradation of the activated NOTCH1 and lead to hyperactivation of the gene.^13,14^ The clinical values of N/Fmut had been reported in T-LBL. NOTCH1 mutations were detected in 55% of T-LBL and relative to better EFS and OS.^13^ N/Fmut was found in 27 of 52 patients with adult T-LBL and confirmed as a favorable prognostic indicator.^15^ Besides, immunohistochemistry of NOTCH1 intracellular domain could help differentiate T-LBL from thymoma.^16^

#### 2.2.2. *PTEN* mutations.

Inactivation of phosphatase and tensin homolog (*PTEN*) gene, a tumor-suppressor gene, occurs in many types of malignancies.^17^ PTEN protein inactivates the PI3K/AKT/mTOR signaling pathway closely related to the development and drug-resistance of tumors by negatively regulating phosphoinositide-3-kinases.^18,19^ Loss function of PTEN results in hyperactivation of the signaling pathway. The deletion of PTEN could induce the development of T-LBL in CD45-expressing mouse.^20^ Moreover, *PTEN* was identified as a poor prognostic marker. The unfavorable prognostic effect may be outweighed in combination with *NOTCH1* mutations.^21,22^ Besides, loss of heterozygosity at chromosome 6q (LOH6q) deletion decreases the poor effect of patients with *PTEN* mutations.^21^

#### 2.2.3. *RAS* mutations.

The RAS/RAF/MEK/ERK signaling pathway has crucial roles in promoting proliferation and survival of cell. Mutations of components of the signaling pathway are frequently founded in various cancers.^23^ *RAS* mutations were detected in 19% of T-ALL patients.^24^ Ksionda et al^25^ found that the overexpression of RasGRP1 made Ras/PI3K/Akt signaling pathway respond to oncogenic cytokines by the increases on basic nucleotide exchange of Ras in T-ALL. Moreover, the RAS/RAF/MEK/ERK pathway was activated by bromodomain-containing protein 2 (BRD2)-mediated direct upregulation of Ras guanyl-releasing protein 1 expression to promote drug resistance in adult T-LBL.^26^

#### 2.2.4. *KMT2D* mutations.

Mutations in *KMT2D* had close relationship with NHL by methylation of transcriptionally active chromatin.^27,28^ One member of SET1 family of histone lysine methyltransferases was encoded by *KMT2D*, which modifies transcriptionally active chromatin by catalyzed methylation of lysine 4 on histone H3 (H3K4).^27^ Loss-of-function mutations in *KMT2D* contribute to tumorigenesis by dysregulating enhancers/super-enhancers-associated genes.^29^ The cumulative incidence of recurrence in patients with *KMT2D*mut was more than that in *KMT2D* wild type, which suggested the status of *KMT2D* was relative to prognosis.^30^ Besides, effects on structure and function were analyzed by structural analysis of mutated domains of *KMT2D*, which provide the evidence of T-LBL tumorigenesis.^30^

#### 2.2.5. Loss of heterozygosity at chromosome 6q.

LOH6q is possibly related to loss of the important tumor-suppressor genes, which have been found in solid tumors and hematologic neoplasms.^31^ LOH6q is detected in 19% of patients with T-LBL, and affects the caspase 8-associated protein 2 gene (*CASP8AP2*).^32^ CASP8AP2 participates in programmed cell death mediated by the FAS signaling pathway and tumor necrosis factor α (TNFα), transcription regulation by c-Myb and corticoid receptor.^33^ Moreover, LOH6q is also correlated with the increased risk of recurrence and unfavorable clinical outcome of T-LBL.^34^

#### 2.2.6. JAK-STAT signaling pathway.

The JAK-STAT signaling pathway has close relationship with proliferation, differentiation and survival of lymphoid precursor cells.^35^ Recruitment of negative regulators and dephosphorylation of JAK2 mediated by protein-tyrosine phosphatases participate in downregulation of the JAK-STAT signaling pathway.^36,37^ Roncero et al^38^ suggested that active JAK2 was involved in the development of T-LBL.

#### 2.2.7. Noncoding RNAs.

Emerging evidences demonstrated that some microRNAs were abnormally expressed during the development and progression of hematological malignancies.^39,40^ MicroRNA-374b suppressed cell proliferation both in vitro and in vivo and promoted cell sensitivity to cytotoxic agent-induced apoptosis by directly targeting AKT1 and Wnt16 in T-LBL. Downregulation of microRNA-374b had a close relationship with the development and drug-resistance of T-LBL.^41^ Additionally, microRNA-211, a tumor suppressor, targets TCF12 to reduce viability and DNA synthesis of T-LBL cells.^42^ But microRNA-21 was upregulated in tumor tissues of T-LBL/ALL as a pro-oncogenic factor.^43^ Besides, maternally expressed gene 3 (MEG3), a long noncoding RNA, suppressed cell migration, invasion, and drug-resistance by suppressing PI3K/mTOR signaling pathway in T-LBL.^44^

## 3. THERAPEUTIC MODALITIES

### 3.1. Chemotherapy

The optimal therapeutic strategies have not been established in T-LBL due to its scarcity and difficulty in differentiating it from T-ALL. Conventional or intensive NHL protocols based on CHOP-like chemotherapy were implemented in T-LBL in early era.^45^ LBL patients treated with CHOP-like regimen achieved CR rate of 55%.^46^ A more intensive protocol improved CR rate to 71%, but the 5-year EFS and OS were 22% and 32%, respectively. These results demonstrated worse clinical outcomes in LBL compared with other aggressive NHL treated with the same protocols^47^ (Table 1).

**Table 1:** Results of various of therapeutic regimens in adult T-LBL.

Authors	Treatment	Lineage	N	Median age (y)	Mediastinal radiotherapy	CNS prophylaxis	CR rate (%)	EFS/DFS/PFS	OS
Kaiser et al^46^	CHOP-like	T+B	29	45	No	No	55	UK	27 mo (median)
Le et al^47^	LNH87/93 trials	T+B (83% T-LBL)	92	31	No	IT MTX in LNH87 protocol	71	22% (5 y)	32% (5 y)
Hoelzer et al^6^	GMALL	T	45	25	24 Gy	24Gy	93	62% (7 y)	51% (7 y)
Thomas et al^7^	Hyper-CVAD	T+B (79% T-LBL)	33	28	30–39 Gy	alternating IT MTX and cytarabine	91	62% for T-LBL (3 y)	70% for T-LBL (3 y)
Li et al^48^	modified NHL-BFM-95	T+B (79% T-LBL)	136	28	No	HD-MTX + IT chemotherapy	77 for T-LBL	48% (5 y)	54% (5 y)
Li et al^48^	hyper-CVAD/MA	T+B (70% T-LBL)	71	32	For mediastinal disease	alternating HD-MTX and cytarabine	64 for T-LBL	26% (5 y)	30% (5 y)
Zheng et al^49^	modified NHL-BFM-90	T+B (97% T-LBL)	30	30	No	IT cytarabine	50	43% (3 y)	46% (3 y)
Lepretre et al^15^	pediatric-like ALL therapy	T	131	33	No	IT chemotherapy and cranial irradiation	91	63% (3-y EFS)	69% (3 y)
Ellin et al^50^	Intensive ALL-type	T	30	40	21–36 Gy	IT chemotherapy	40	49% (5 y)	48% (5 y)
Cortelazzo et al^51^	NILG-ALL 09/00	T	24	32	24 Gy	CNS chemo-radioprophylaxis	92	71% (5 y)	69% (5 y)

CNS = central nervous system, CR = complete remission, DFS = disease-free survival, EFS = event-free survival, HD = high dose, IT = intrathecal, LBL = lymphoblastic lymphoma, MTX = methotrexate, OS = overall survival, PFS = progression-free survival, UK, unknown.

ALL-like treatment regimens have been recommended for adult T-LBL (Table 1). These protocols typically consisted of multidrug induction treatment (often including cyclophosphamide, anthracycline, vincristine, and steroid), consolidation treatment, and central nervous system (CNS) prophylaxis. Forty-five adult T-LBL patients were treated with ALL-like regimens (GMALL 04/89 protocol or GMALL 05/93 regimen) combining with prophylactic brain and mediastinal radiotherapy with 62% and 51% of 7-year disease-free survival (DFS) and OS, respectively.^6^

Intensive or modified hyper-CVAD regimens including cyclophosphamide, vincristine, anthracycline, and dexamethasone was successful therapeutic strategies for adult T-LBL (Table 1). Thirty-three patients with LBL (79% T-LBL) accepted standard or modified hyper-CVAD regimens with a CR rate of 91%, and the estimated 3-year progression-free survival (PFS) and OS were 62% and 67%, respectively.^7^

Furthermore, adult T-LBL treated with modified NHL-Berlin-Frankfurt-Müster-95 (NHL-BFM95) had higher CR and minimal residual disease (MRD) rate (77% vs 64%, 69% vs 53%, respectively) and more favorable survival than those received hyper-CVAD.^48^ Similar results were reported in modified NHL-BFM90 for adult T-LBL with CR, 3-year PFS and OS of 50%, 43%, and 46%, respectively.^49^ These findings suggested that modified NHL-BFM90 and NHL-BFM95 were alternative therapeutic strategies for adult T-LBL (Table 1).

Superior OS in adults ALL treated with pediatric-inspired ALL protocols were reported in several studies, which shed new light on treating adult T-LBL.^52,53^ The prospective clinical trial on the efficacy of pediatric-like ALL protocol for adult T-LBL reported 91%, 63%, 72%, and 69% of CR, 3-year EFS, DFS, and OS, respectively^15^ (Table 1). These findings rationalized the application of pediatric-like ALL regimen for adult T-LBL.

### 3.2. Mediastinal radiotherapy

Though T-LBL often presented a mediastinal mass, mediastinal radiotherapy remains controversial. Cortelazzo et al^51^ reported that the mediastinal relapse rate was 7% in 14 nonradiotherapy patients with T-LBL. However, among the 34 relapsed adult T-LBL in the GRAALL-LYSA LL03 study, 14 (41%) patients recurred in the mediastinum. This result promoted clinicians to consider using local mediastinal radiotherapy to improve the possibility of CR.^15^

Hoelzer et al^6^ observed that 6 patients had received mediastinal irradiation in the 7 adult T-LBL with mediastinal recurrence, and concluded that local mediastinal irradiation did not decrease the risk of mediastinal relapse. In the meantime, the adverse effects induced by mediastinal irradiation such as radiation pneumonitis,^54^ cardiac injury,^55^ thyroid dysfunction,^56^ and second malignancy,^57^ restricted the application of mediastinal radiotherapy. Another study reported that the mediastinal radiation therapy could significantly decrease the risk of mediastinal relapse, but not affect the OS of adult T-LBL.^58^ Notably, the adult T-LBL patients treated with hyper-CVAD in combination with mediastinal radiotherapy had no local recurrence and prolonged the time to progression.^50^ Overall, mediastinal radiotherapy could improve the local control rate and DFS.

Because of the relatively high risk of recurrence in patients with T-LBL, patients would possibly receive high-dose therapy protocols after their recurrence. Hence, it is critical to avoid excess radiation-related adverse events by optimizing radiotherapy techniques, radiation fields, target volumes, and radiation dose.

### 3.3. CNS prophylaxis

The invasion of CNS is relatively low in patients with LBL at diagnosis, ranging from 3% to 9%.^45^ However, CNS is the frequently recurred site if CNS prophylaxis is not included in the therapeutic protocol. About 40% to 100% of CNS relapse occurred in patients treated with NHL-type regimens without CNS-treatment.^59^ The incidence of CNS recurrence was decreased to 3% in patients treated with CNS prophylaxis.^60^ Consequently, CNS prophylaxis is essential for patients with T-LBL.

Prophylactic cranial irradiation (PCI) can induce cognitive and neurosensory impairment, cerebrovascular disease, and even second neoplasm, which have unfavorable effects on the quality of life of patients.^61,62^ These adverse events restricted the application of PCI as CNS prophylaxis for patients. A successful clinical trial indicated that intensifying high-dose methotrexate (HD-MTX, 5 g/m^2^) without cranial irradiation can achieve a low rate of CNS relapse (1/105).^63^ Additionally, a cumulative rate of CNS recurrence was 1.2% in a trial by intensifying intrathecal MTX without cranial irradiation.^5^ Intensive ALL-like therapy in combination with intrathecal MTX was adequate for CNS prophylaxis with no CNS recurrence.^64^ Also, the combination of HD-MTX with intrathecal cytarabine was proper.^65^ Therefore, CNS prophylaxis should be routinely applied in T-LBL, and radiation-free protocols are proper approaches.

### 3.4. Hematopoietic stem cell transplantation

There was a controversy about the use of HSCT as a consolidation therapy after induction chemotherapy and early remission. The estimated 2-year DFS and OS were 77% and 85% for adult LBL treated with autologous HSCT (auto-HSCT) after their first CR (CR1),^66^ and the 3-year EFS and OS were 31% and 48% in another study.^67^ Adult LBL treated with allogeneic HSCT (allo-HSCT) obtained 5-year EFS and OS of 45% and 49%, respectively.^68^ But there was no significant difference between allo- and auto-HSCT in 5-year lymphoma-free survival (36% vs 39%, *P* = 0.82).^69^ These findings in adult LBL were not more favorable than those treated with intensifying ALL-like protocols.^70,71^

HSCT is recommended for adult T-LBL with unfavorable prognostic factors after their CR1.^72^ In the NILG study, adult LBL with poor response and/or MRD+ received HSCT based on a risk-adapted strategy with 5-year DFS and OS of 77% and 72%, respectively.^51^ With respect to relapsed/refractory (r/r) LBL, they had extremely poor OS, only a few achieved a long-time survival after receiving HSCT.^8^ The therapeutic strategy is to implement HSCT following by a new remission after new induction therapy for r/r LBL.^45^

### 3.5. New treatment and drugs

#### 3.5.1. Chimeric antigen receptor-T-cell immunotherapy.

Chimeric antigen receptor (CAR) immunotherapy is a revolutionary treatment in hematological malignancies, especially CD19 CARs in B-cell malignancies.^73^ The success of CD19 CARs in treating CNS diffuse large-B-cell lymphoma demonstrated CAR T cells could go through the blood–brain barrier.^74^ CD5 is one of the surface antigens expressed on T-cell malignancies but not hematopoietic stem cells.^75^ A relapsed T-LBL patient with CNS involvement improved symptoms and remitted disease after treatment with CD5-IL15/IL15sushi CAR-T-cell immunotherapy. And the lymphoblastic cells in cerebrospinal fluid was rapidly depleted.^76^ These findings suggest that CD5 CAR-T-cell immunotherapy could be a novel therapeutic regimen for r/r T-LBL. Besides, the clinical study of CD7 CAR-T-cell immunotherapy in treatment of r/r T-LBL or T-ALL was recruiting (NCT04916860). More clinical trials about CAR-T therapy were listed in Table 2.

**Table 2:** Information about some clinical trials of adult T-LBL which were recruiting (www.clinicaltrials.gov).

ClinicalTrials.gov identifier	Intervention	Condition(s)	Age, y	Phase
NCT04916860	CD7 CAR-T cells	CD7+ r/r T-ALL/LBL	2–70	UK
NCT04934774	CD7 CAR-T cells	r/r T cell malignances	18 or more	Phase 1
NCT04004637	CD7 CAR-T cells	CD7+ r/r T-ALL/LBL or NK/T cell lymphoma	7–70	Phase 1
NCT04984356	CD7 allogeneic CAR-T cells	r/r T-ALL/LBL	12 or more	Phase 1/2
NCT04594135	CD5 CAR-T cells	r/r T cell malignances	8 or more	Phase 1
NCT03690011	CD7.CAR/28zeta CAR-T cells	recurrent T cell malignances	up to 75	Phase 1
NCT04446130	Decitabine combined with HAAG regimen	newly diagnosed ETP-ALL/LBL, T/M-MPAL, T-ALL/LBL	15–60	Phase 3
NCT03328104	Everolimus combined with nelarabine, cyclophosphamide and etoposide	r/r lymphoblastic leukemia/lymphoma	2–29	Phase 1
NCT00501826	Hyper-CVAD plus nelarabine	previously untreated T-ALL/LBL	Child, adult, and older adult	Phase 2
NCT02763384	BL-8040 and nelarabine	r/r T-ALL/LBL	18 or more	Phase 2
NCT05032183	Tagraxofusp and low-intensity chemotherapy	CD123+ r/r lymphoblastic leukemia/lymphoma	18–70	Phase 1/2

CAR = chimeric antigen receptor, ETP-ALL/LBL = early T-cell precursor lymphoblastic leukemia/lymphoma, r/r = relapsed/refractory, T-ALL/LBL = T-cell lymphoblastic leukemia/lymphoma, T/M-MPAL = T-cell/myeloid mixed phenotype acute leukemia, UK = unknown.

#### 3.5.2. *NOTCH1* inhibitors.

Because *NOTCH1* mutations play critical pathogenetic roles in T-LBL/ALL, *NOTCH1* inhibitors may be favorable drugs for the therapy of T-LBL/ALL. Gamma secretase inhibitors (GSI) exert their antitumor activity by blocking *NOTCH1* activation. In a phase I clinical trial, one of 25 patients with r/r T-LBL/ALL achieved CR after treatment with BMS-906024 GSI. All of 13 evaluable patients had stable disease with the median PFS and OS of 9.9 and 47.3 months in subsequent phase II trial.^77^ This treatment represents a new thought of therapeutic strategy for T-LBL.

#### 3.5.3. Cytotoxic agents.

Nelarabine shows toxicity to T-cell by inhibiting synthesis of DNA as a deoxyguanosine analog, and cannot be degraded by the purine nucleoside phosphorylase. It was approved for the therapy of r/r T-ALL/T-LBL by the US Food and Drug Administration.^78^ Adult T-ALL/T-LBL who were r/r to 2 or more induction therapy regimens achieved the CR rate of 21%.^78^ Similarly, CR rate was 36% for r/r T-ALL/T-LBL who accepted nelarabine as savage therapy, and the estimated 2- and 5-year OS were 46% and 38% for 47 patients who received HSCT after nelarabine therapy.^79^ Furthermore, the CR rate of T-LBL was 100%, and the 3-year OS was 65% in adult T-LBL/ALL who were treated with hyper-CVAD plus nelarabine as first-line therapy. But survival benefit was not obtained for patients who received this combination, compared to historic hyper-CVAD data of Abaza et al^80^ The optimal schedule for combining nelarabine with multiagent chemotherapy regimens remains a hot topic.

Clofarabine, a purine nucleoside analog, was approved in salvage therapy for r/r ALL. Among 31 patients with r/r LBL/ALL treated with clofarabine-based therapy, 31% had CR and the 1-year OS was 10%.^81^ It deserves assessment in patient with T-LBL.

Clinical trials of combination treatment were listed in Table 2.

## 4. PROGNOSTIC MODELS

Few clinical features of adult T-LBL such as performance status (PS), age, BM involvement, CNS involvement, lactate dehydrogenase (LDH) have been indicated as prognostic factors.^7,70,82,83^ These features were not consistent in distinct studies.^7,84^ It was known that immunophenotypic subtypes of adult T-LBL have prognostic value. Early T-cell precursor (ETP) ALL/LBL is a high-risk subset of adult T-ALL/LBL, which has poor long-term clinical outcomes.^85^ In general, clinical features of adult T-LBL are insufficient for prognostic prediction and clinical decision-making.

The genetic and molecular factors have been explored for prediction of prognosis and clinical decision-making. *N*/F mutations had favorable prognostic effect in T-LBL.^86^ Minimal disseminated disease combining with *N/F* mutations could help identify patients benefit from intensive treatment regimens.^87^ The 4-gene classifier (*NOTCH1/FBXW7/RAS/PTEN*) was considered as an independent prognostic indicator for EFS, DFS, and OS in adult T-LBL.^15^ In addition, Tian et al^88^ developed a five-miRNAs-based classifier to predict prognosis for adult T-LBL after CR1. Patients with high risk of this classifier could benefit from HSCT after CR1, especially combining with PS and *NOTCH1*/*FBXW7* status, which can help personalized treatment for adult T-LBL.^88^ An 11-gene-based classifier was developed to predict clinical survival outcomes of adult T-LBL. A nomogram prognostic model consisting of the 11-gene-based classifier, PS, NOTCH1/FBXW7 status, LDH, and CNS involvement enhanced the prognostic accuracy. And adult T-LBL patients with high nomogram score could benefit from the BFM regimen, which may contribute to precision therapy decision-making.^89^ Moreover, a 4-CpG-based classifier showed a good predictive effect in adult T-LBL, and a prognostic model incorporating this classifier, PS, *NOTCH1*/*FBXW7* status, LDH, and CNS involvement helped identify high-risk subgroups of adult T-LBL mostly benefited from HSCT. The therapeutic protocol with BFM followed by HSCT could significantly improve survival of high-risk adult T-LBL.^90^

## 5. DISCUSSION AND CONCLUSIONS

Genetic and molecular alternations were explored in adult T-LBL, but the internal mechanism of action and large-scale research was warranted. To date, ALL-type or pediatric-inspired ALL protocols are implemented in the treatment of adult T-LBL. Mediastinal radiotherapy improved local control and EFS, but adverse events induced by mediastinal irradiation are of concern. CNS prophylaxis is critical for adult T-LBL, and cranial radiation-free CNS prophylaxis is more appropriate than PCI. Some prognostic models helped identify high-risk patients benefited from a specific intensive induction therapeutic protocol or HSCT after CR1. HSCT is recommended for high-risk adult T-LBL after CR1 and r/r adult T-LBL. Exploring new treatment and novel drug is needed.
